# Long-term outcome of vagus nerve stimulation therapy in drug-resistant epilepsy: a retrospective single-center study

**DOI:** 10.3389/fneur.2025.1564735

**Published:** 2025-05-09

**Authors:** Si Chen, Xiaxin Yang, Shujun Xu, Baomin Li, Chao Li, Ning Yang, Xue Yang, Xiaotang Wang, Shuo Xu, Xiuhe Zhao

**Affiliations:** ^1^Department of Neurology, Qilu Hospital of Shandong University, Cheeloo College of Medicine, Shandong University, Jinan, Shandong, China; ^2^Department of Neurosurgery, Qilu Hospital of Shandong University, Cheeloo College of Medicine and Institute of Brain and Brain-Inspired Science, Shandong University, Jinan, Shandong, China; ^3^Department of Pediatrics, Qilu Hospital of Shandong University, Cheeloo College of Medicine, Shandong University, Jinan, Shandong, China; ^4^National Medicine-Engineering Interdisciplinary Industry-Education Integration Innovation Platform, Shandong Key Laboratory:Magnetic Field-free Medicine & Functional Imaging, Research Institute of Shandong University:Magnetic Field-free Medicine & Functional Imaging, Jinan, Shandong, China

**Keywords:** epilepsy, vagus nerve stimulation, drug-resistant epilepsy, prognostic factors, rapid response

## Abstract

**Introduction:**

Vagus nerve stimulation (VNS) has garnered widespread application in patients with drug-resistant epilepsy (DRE), while the efficacy and prognostic factors of VNS in DRE remain elusive. Moreover, clinical determinants associated with rapid response to VNS have never been uncovered. This study aimed to elucidate factors influencing efficacy and rapid response to VNS.

**Methods:**

A consecutive series of patients with DRE undergoing VNS surgery from January 2014 to December 2023 was collected to describe VNS efficacy. Both univariate and multivariate analyses were performed to identify statistically significant prognostic factors, and a predictive model was developed. Furthermore, we examined clinical determinants of rapid/slow response to VNS and VNS current changes.

**Results:**

A total of 65 patients underwent VNS implantation. Seizure frequency significantly decreased post-VNS, with mean seizure reduction rates of 35.7, 49.0, 48.5, 52.8, 63.2, and 66.8% at 6 (*n* = 65), 12 (*n* = 65), 24 (*n* = 50), 36 (*n* = 40), 60 (*n* = 31), and 84 (*n* = 19) months, respectively. At final follow-up, 61.5% (40/65) were responders (50–100% seizure reduction), and 10.8% (7/65) achieved seizure freedom for ≥1 year. Univariate analysis identified age at seizure onset ≥6 years (*p* = 0.003), baseline seizure frequency ≤30/month (*p* = 0.001), focal seizures (*p* = 0.002), developmental and epileptic encephalopathies (*p* = 0.037), and surgical history (*p* < 0.001) as significant prognostic factors. Multivariate analysis confirmed age at seizure onset ≥6 years (OR: 5.726, *p* = 0.039), baseline seizure frequency ≤30/month (OR: 4.697, *p* = 0.048), and focal seizures (OR: 4.791, *p* = 0.025) as independent predictors, enabling the development of a predictive model for VNS efficacy. Additionally, among responders, the median response duration was 6 months (range: 1–60 months), with baseline seizure frequency ≤30/month significantly associated with rapid response of VNS in DRE (<6 months, *p* = 0.033).

**Conclusion:**

Vagus nerve stimulation is effective for treating DRE, with efficacy increasing with follow-up duration. Age at seizure onset ≥6 years, baseline seizure frequency ≤30/month, and focal seizure were predictive of VNS success, underscoring the need for careful preoperative assessment of patients with DRE before VNS surgery.

## 1 Introduction

Epilepsy is one of the most common chronic neurological disorders, presenting with numerous comorbidities such as cognitive disability and depression, which significantly impair patients’ quality of life. Despite the approval and routine use of novel antiseizure medications (ASMs), up to a third of patients with epilepsy remain unable to achieve seizure freedom (1, 2), which is defined as drug-resistant epilepsy (DRE), the failure to achieve sustained seizure freedom after adequate trials of two tolerated, appropriately chosen, and used antiepileptic drug treatments, whether as monotherapies or in combination (3, 4). Patients with DRE face not only complex medical challenges but also substantial social, psychological, and economic burdens.

In recent years, patients with DRE have benefited from various neurostimulation techniques, including vagus nerve stimulation (VNS), deep brain stimulation, and closed-loop-responsive neurostimulation (5). Among these techniques, VNS has garnered widespread attention due to its minimal invasiveness and high safety profile, particularly in patients with an unclear epileptogenic focus or those with difficulty in seizure control after lesion resection. In 2022, two meta-analyses published by the International League Against Epilepsy (ILAE) on neuromodulation therapies indicated that, for generalized epilepsy with a follow-up of 39.1 months, VNS resulted in a 48.3% reduction in seizure frequency; moreover, for DRE, with an average follow-up of 1.3 years, the reduction in seizure frequency was 34.7% (5, 6). However, in long-term follow-up studies, Wang et al. observed that 82.9% of patients who received treatment for 12 years experienced a reduction in seizure frequency of at least 50% (7), suggesting the vital role of long-term follow-up in assessing VNS efficacy. Given the variability in seizure outcomes following VNS therapy, previous studies have sought predictive factors for VNS efficacy (8–10). A systematic review identified several promising biological biomarkers of VNS efficacy in DRE, the routine clinical application of these biomarkers remains limited by practical constraints (11). Nonetheless, the limited number of subjects and short follow-up time in these studies hindered further analyses, especially regarding differences between adults and children and clinical determinants of rapid response to VNS. Therefore, unraveling the clinical characteristics and associated prognostic factors of VNS in DRE with a larger sample size and longer follow-up is urgently needed. This effort will provide neurologists with valuable insights to improve the evaluation of patient’s clinical conditions and enhance understanding of the association between epileptic seizures and VNS efficacy in DRE.

In this retrospective analysis, we reviewed the demographic and clinical features of 65 patients who underwent VNS surgery at our center to analyze the prognostic factors for VNS effectiveness and develop a prediction model of its efficacy in patients with DRE.

## 2 Methods

### 2.1 Study design and participants

This retrospective, observational, and descriptive study was conducted at Qilu Hospital of Shandong University, involving patients with epilepsy who received VNS between January 2014 and December 2023. In our comprehensive epilepsy center, we generally follow the guidelines outlined in the Chinese consensus on VNS for the treatment of DRE (12, 13) and the VNS guidelines reported by the Guideline Development Subcommittee of the American Academy of Neurology (14). The criteria for VNS implantation are as follows: (1) patients must meet the diagnostic criteria for DRE as defined by the ILAE (3). (2) A comprehensive anatomo-electro-clinical evaluation, including but not limited to semiology, EEG, MRI, PET-CT, etc., is performed to exclude any treatable cause, or in cases of whom previous therapeutic attempts have failed. In some rare cases, patients may opt for VNS therapy due to the involvement of the epileptogenic zone in eloquent areas of the cortex, or because they have chosen not to undergo epilepsy surgery after careful consideration. Before the VNS implantation, all patients undergo a thorough multi-disciplinary evaluation. The patients who met the aforementioned inclusion criteria underwent VNS implantation (PINS Medical, Ltd., Beijing, China; http://www.pinsmedical.com/). Similar to the LivaNova VNS system, the PINS device consists of key components including a pulse transmitter, stimulation electrode, and a wearable magnet.

The study was approved by the Ethics Committee of Qilu Hospital of Shandong University. All methods were in accordance with relevant guidelines and regulations. Written informed consent was obtained from all patients or their guardians prior to participation.

### 2.2 Clinical data

Demographic and clinical data, including sex, age at seizure onset, age at VNS, disease duration, seizure frequency, seizure type (focal or generalized), seizure symptoms (motor or non-motor), developmental and epileptic encephalopathies (DEEs), etiology, number of ASMs, and surgical history, were collected at baseline and during follow-up. Preoperative brain MRI, video EEG, and postoperative VNS stimulation settings were also obtained. Importantly, the seizure frequency of all patients at different follow-up times was recorded to calculate the seizure reduction rate, response rate and seizure freedom rate for at least 1 year.

To investigate the prognostic factors of VNS, postsurgical seizure outcomes were evaluated according to the modified McHugh classification: Class I (80–100% reduction in seizure frequency), Class II (50–79% reduction), Class III (<50% reduction), Class IV (beneficial only when using a magnet), and Class V (no improvement). Patients were classified into two groups: Group 1 (responders) and Group 2 (non-responders). Classes I–II were defined as responders, while classes III–V indicated non-responders. To identify clinical factors associated with rapid response to VNS efficacy, the responder group were further divided into rapid and slow responders, according to the median response time of the responders. The study aims to evaluate the efficacy of VNS in reducing seizure frequency and identify prognostic factors associated with VNS treatment response.

### 2.3 VNS programming procedure

According to VNS guidelines for DRE in China (12, 13), the VNS treatment system initiated neurostimulation following a 1–2 weeks start-up period. Initial device parameters were typically programmed as follows: output current (0.2–0.5 mA), frequency (30 Hz), pulse width (250–500 μs), stimulation duration (30 s), and inter-stimulus interval (5.0 min). The protocol consisted of two distinct phases. Phase I (Dose Titration): For tolerant patients, the output current may be incrementally increased by 0.2–0.5 mA every 2 weeks. Through this gradual titration process, most patients achieved therapeutic current levels (1.0–1.5 mA) within 8–12 weeks. Phase II (Maintenance Therapy): subsequent parameter adjustments were performed quarterly, with modifications based on comprehensive evaluation of both seizure frequency and patient tolerance.

### 2.4 Statistical analysis

Statistical analyses were performed using SPSS 26.0 (IBM Corp., Armonk, NY, United States) and R 4.3.1 (University of Auckland, Auckland, New Zealand) software. Clinical characteristics were presented as the mean ± standard deviation (SD) (range) or median (interquartile range, IQR) for continuous data and *n* (%) for categorical data. Statistical tests were assessed with the Pearson *χ*^2^ test or Mann–Whitney *U* test, as appropriate. Binary logistic regression analysis was employed to evaluate prognostic factors for VNS efficacy in DRE. Additionally, a nomogram model was developed to predict responses to VNS in patients with DRE based on the results of binary logistic regression analysis. The receiver operating characteristic (ROC) curve, calibration plots, and clinical decision curves (DCAs) were employed to evaluate the prediction model. *p* < 0.05 was set as the threshold for statistical significance across all analyses.

## 3 Results

### 3.1 Demographic and clinical features of DRE patients

Clinical data were collected from 65 patients with DRE implanted with VNS, including 40 male and 25 female subjects (Table 1). The median age at seizure onset was 3.0 years (IQR 1.0–7.8 years), and the median age at VNS implantation was 9.8 (IQR 6.0–19.0 years), with 46 children and 19 adults. The median duration from seizure onset to VNS implantation was 6.0 years (IQR 3.1–10.3 years). Among the 65 patients with DRE who underwent VNS, focal seizure was the most common type (60.0%, 39/65), followed by generalized seizure (40.0%, 26/65). Approximately 66.2% (43/65) of subjects presented with motor seizures only, while the others presented with both motor and non-motor seizures. Regarding the etiology, 26.2% (17/65), 12.3% (8/65), 13.8% (9/65), 1.5% (1/65), and 46.3% (30/65) had structural, genetic, infectious, immune, and unknown causes, respectively. Additionally, 20.0% (13/65) and 40.0% (26/65) had temporal epilepsy and DEEs, respectively.

**Table 1:** Demographic and clinical characteristics of 65 DRE patients with VNS.

Variable	Total (*n* = 65)
Gender (male:female), *n*	40:25
Age at seizure onset, IQR (years)	3.0 (1.0, 7.8)
Age at VNS, IQR (years)	9.8 (6.0, 19.0)
Age at VNS < 18 years, *n* (%)	46 (70.8)
Disease duration, IQR (years)	6.0 (3.1, 10.3)
Baseline seizure frequency per month, IQR	30.0 (6.0, 112.5)
Responder, *n* (%)	40 (61.5)
Seizure free, *n* (%)	7 (10.8)
Temporal epilepsy, *n* (%)	13 (20.0)
Seizure type, *n* (%)	
Focal	39 (60.0)
Generalized	26 (40.0)
Seizure symptom, *n* (%)	
Only motor	43 (66.2)
Only non-motor	0 (0.0)
Motor and non-motor	22 (33.8)
DEEs, *n* (%)	26 (40.0)
Etiology, *n* (%)	
Structural	17 (26.2)
Genetic	8 (12.3)
Infectious	9 (13.8)
Immune	1 (1.5)
Unknown	30 (46.2)
EEG epileptiform discharge, *n* (%)	
Generalized	25 (38.5)
Multi	30 (46.2)
Focal	10 (15.4)
Number of ASMs before VNS, IQR	3 (3, 4)
Number of ASMs after VNS, IQR	3 (2, 3)
Positive MRI, *n* (%)	30 (46.2)
Operation history, *n* (%)	8 (12.3)
VNS current, IQR (mA)	1.6 (1.3, 1.8)
Follow-up duration, IQR (month)	40 (24, 84)

DRE, drug-resistant epilepsy; VNS, vagus nerve stimulation; IQR, interquartile range; DEEs, developmental and epileptic encephalopathies.

All patients with available EEG recordings showed abnormal epileptiform discharge, as shown in Table 1. Generalized, multiple, and focal discharge was observed in 38.5% (25/65), 46.2% (30/65), and 15.4% (10/65) of patients, respectively.

### 3.2 Seizure outcomes of DRE patients with VNS

**Figure 1:**
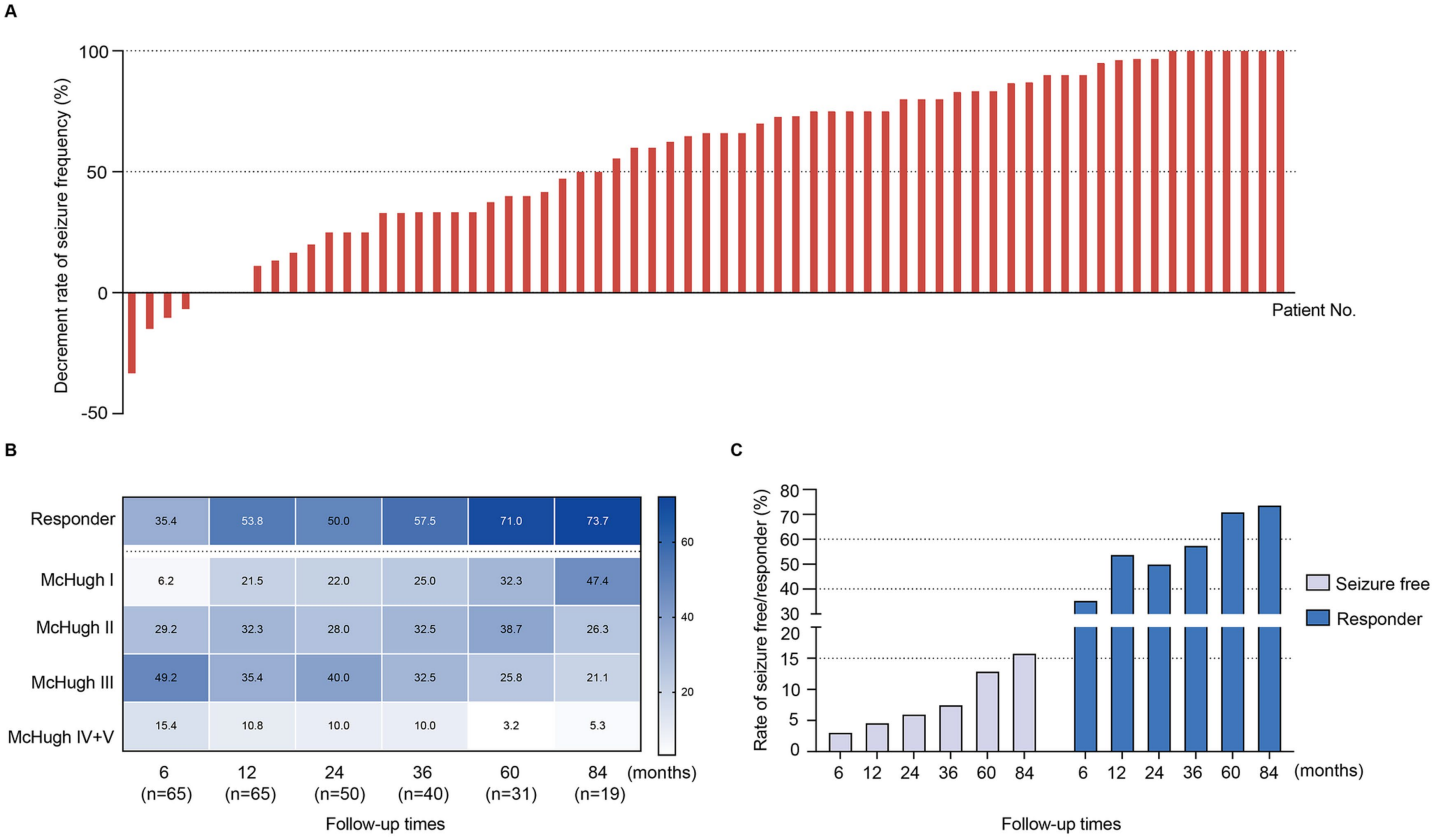
The seizure outcomes of DRE patients with VNS. **(A)** Decrement rate of 65 DRE patients with VNS at final follow-up. **(B)** The percentage of responders and McHugh I/II/III/IV + V at different follow-up times after VNS. **(C)** The percentage of seizure freedom and responder at different follow-up times after VNS.

The median follow-up duration were 40 months (12 months to 120 months, IQR 24 months to 84 months). The follow-up periods were 6 (*n* = 65), 12 (*n* = 65), 24 (*n* = 50), 36 (*n* = 40), 60 (*n* = 31), and 84 (*n* = 19) months. Seizure outcomes for the 65 patients at the last follow-up were shown in Figure 1A. Overall, 65 patients experienced a mean reduction in seizure frequency of 56.7 ± 34.7% with a median follow-up of 40 months. Moreover, a decrease in seizure frequency of greater than 50.0% was observed in 61.5% (40/65) of all patients, and seizure freedom for at least 1 year was achieved in 10.8% (7/65) patients. The mean reduction in seizure frequency at 6, 12, 24, 36, 60 and 84 months were 35.7, 49.0, 48.5, 52.8, 63.2, and 66.8%, respectively.

According to the modified McHugh classification, 61.5% of patients were classified as responders (50–100% reduction in seizure frequency), including 10.8% seizure-free, while the remaining were non-responders (<50% reduction in seizure frequency) (Figure 1A and Table 1). The detailed rates of VNS responders across follow-up periods are illustrated in Figures 1B,C. At 6, 12, 24, 36, 60, and 84 months, the proportions of VNS responders were 35.4% (23/65), 53.8% (35/65), 50.0% (25/50), 57.5% (23/40), 71.0% (22/31), and 73.7% (14/19), respectively, which were gradually upregulated (Figures 1B,C); similar trends were obtained in the seizure-free group, with proportions of 3.1% (2/65), 4.6% (3/65), 6.0% (3/50), 7.5% (3/40), 12.9% (4/31), and 15.8% (3/19), respectively.

The median number of preoperative ASMs was 3 (IQR 3–4). After VNS, the median number of ASMs decreased to 3 (IQR 2–3), as shown in Table 1. Notably, there was a highly significant reduction in the number of ASMs post-VNS compared to the pre-procedure state (*p* = 0.0003). During the long-term follow-up period after the operation, 4.62% (3/65) patients (4.62%) additionally incorporated a ketogenic diet into their treatment regimen. VNS was relatively safe with an incidence of short-term and reversible adverse reactions of 9.2% (6/65), including coughing (2/65, 3.1%), nausea (1/65, 1.5%) and transient pain (3/65, 4.6%). No patient experienced long-term adverse effects in our cohort.

### 3.3 Analysis of the prognostic factors of VNS efficacy in patients with DRE

To investigate the clinical prognostic factors for VNS efficacy in DRE, the patients were divided into two groups: the responder group (Class I–II) and the non-responder group (Class III–V), according to the modified McHugh classification. Univariate analysis revealed that focal seizure type was more prevalent in the responder group than in the non-responder group (*p* = 0.002). Additionally, baseline seizure frequency ≤ 30/month was closely associated with positive response to VNS (*p* = 0.001; Table 2), indicating that patients with DRE and focal seizures with low seizure frequency are more sensitive to VNS implantation.

**Table 2:** Univariate analysis of clinical variables associated with VNS efficacy in DRE.

Variable	Responder (*n* = 40)	Non-responder (*n* = 25)	*p*
Gender (male:female), *n*	25:15	15:10	0.840
Age at seizure onset, years	3.7 (1.7, 8.5)	1.8 (0.8, 5.1)	0.050
Age at seizure onset ≥6 years, *n* (%)	19 (47.5)	3 (12.0)	**0.003**
Age at VNS, years	10.4 (6.0, 20.5)	9.8 (7.3, 15.0)	0.458
Disease duration, years	5.9 (3.0, 10.3)	6.9 (4.0, 10.0)	0.656
Disease duration ≤2 years, *n* (%)	7 (17.5)	3 (12.0)	0.807
Seizure frequency before VNS, monthly	13.0 (5.0 ~ 60.0)	90.0 (19.0 ~ 150.0)	**0.003**
Baseline seizure frequency ≤30 monthly, *n* (%)	27 (67.5)	6 (24.0)	**0.001**
Seizure type, *n* (%)			**0.002**
Focal	30 (75.0)	9 (36.0)	
Generalized	10 (25.0)	16 (64.0)	
Seizure symptom, *n* (%)			0.407
Only motor	28 (70.0)	15 (60.0)	
Motor and non-motor	12 (30.0)	10 (40.0)	
DEEs, *n* (%)	12 (30.0)	14 (56.0)	**0.037**
Temporal epilepsy, *n* (%)	9 (22.5)	4 (16.0)	0.524
Etiology, *n* (%)			0.362
Structural	12 (30.0)	5 (20.0)	
Genetic	4 (10.0)	4 (16.0)	
Infectious	7 (17.5)	2 (8.0)	
Immune	0 (0.0)	1 (4.0)	
Unknown	17 (42.5)	13 (52.0)	
EEG epileptiform discharge, *n* (%)			0.716
Generalized	14 (35.0)	11 (44.0)	
Multi	20 (50.0)	10 (40.0)	
Focal	6 (15.0)	4 (16.0)	
Number of ASMs before VNS	3 (3,4)	3 (3,4)	0.729
Positive MRI, *n* (%)	18 (45.0)	12 (48.0)	0.813
Operation history, *n* (%)	5 (12.8)	3 (12.0)	**<0.001**
VNS current, mA	1.6 (1.2 ~ 1.8)	1.7 (1.6 ~ 1.8)	**0.032**

DRE, drug-resistant epilepsy; VNS, vagus nerve stimulation; DEEs, Developmental and epileptic encephalopathies. Bold values, statistical significance (*p* < 0.05).

Furthermore, patients with age at seizure onset ≥ 6 years exhibited better prognosis than did those with seizure onset < 6 years (*p* = 0.003); moreover, patients with DEEs showed reduced responsiveness to VNS implantation (*p* = 0.037). Other factors, such as etiology, neuroimaging findings, disease duration, and the number of ASMs, had no significant correlation with VNS efficacy (Table 2).

Multivariate logistic regression analyses identified focal seizure type, age at seizure onset ≥ 6 years, and baseline seizure frequency ≤ 30/month as significant prognostic factors for VNS efficacy (Table 3). Focal seizure increased the likelihood of a positive VNS outcome by 4.791 times compared to generalized seizure [95% confidence interval (CI), 1.213–18.918, *p* = 0.025; Table 3]. Similarly, age at seizure onset ≥ 6 years [odd ratio [OR], 5.726; 95% CI, 1.089–30.098, *p* = 0.039] and baseline seizure frequency ≤ 30/month (OR, 4.697; 95% CI, 1.016–21.719, *p* = 0.048) were positively associated with VNS efficacy in DRE (Table 3).

**Table 3:** Multivariate analysis of clinical variables associated with VNS efficacy in DRE.

Variable	OR	95% Confidence interval	*p*
Age at seizure onset ≥ 6 (years)	5.726	1.089–30.098	**0.039**
Age at VNS < 6 years	1.988	0.428–9.226	0.380
Disease duration ≤ 2 years	0.966	0.133–7.023	0.973
Baseline seizure frequency ≤ 30 monthly	4.697	1.016–21.719	**0.048**
Seizure type (focal vs. Generalized)	4.791	1.213–18.918	**0.025**
DEEs	0.477	0.089–2.551	0.387

DRE, drug-resistant epilepsy; VNS, vagus nerve stimulation; DEEs, Developmental and epileptic encephalopathies. Bold values, statistical significance (*p* < 0.05).

### 3.4 Construction of the nomogram model to predict VNS efficacy

**Figure 2:**
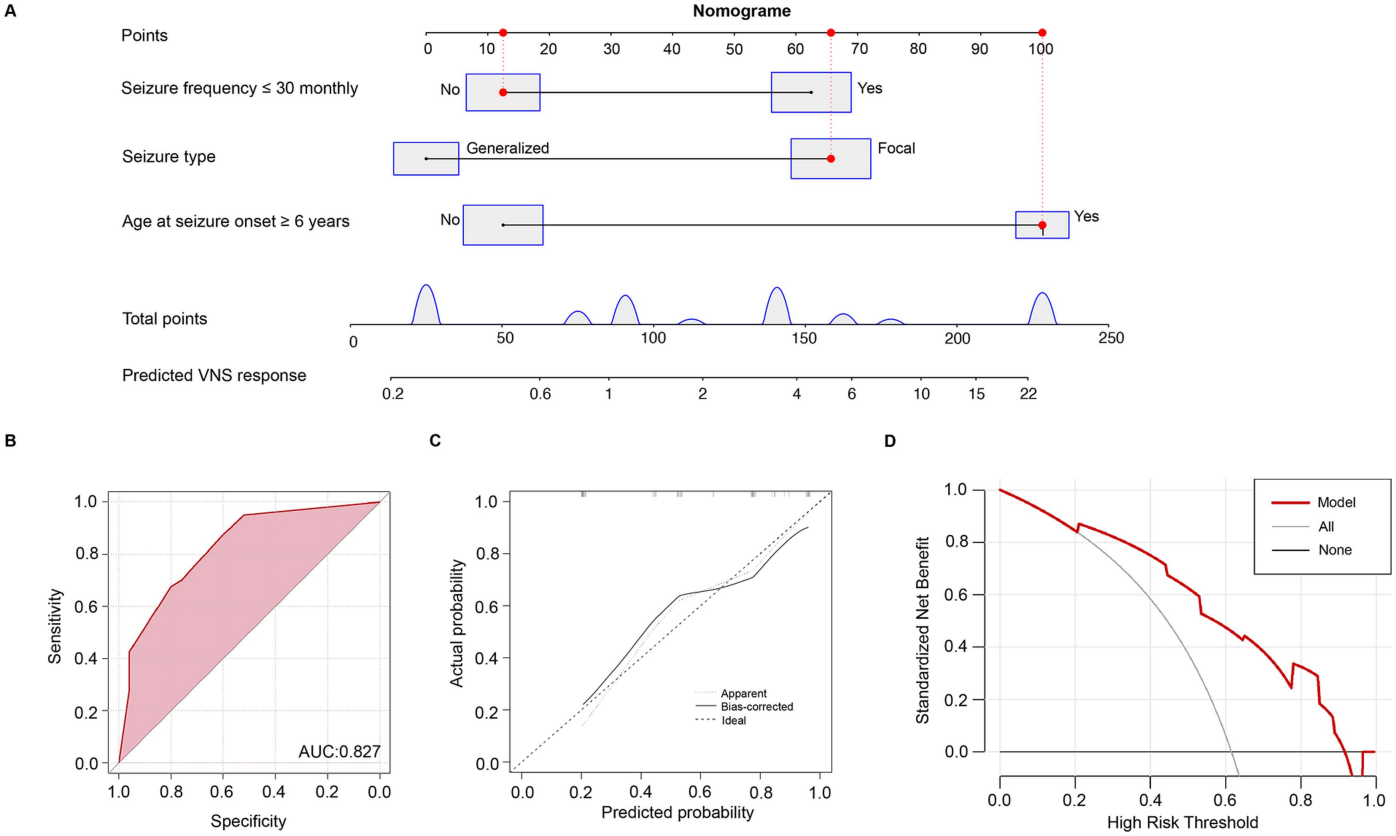
Nomogram model predicting the prognosis of VNS in patients with DRE. **(A)** Nomogram model. **(B)** Receiver operating characteristic curve (ROC) of the nomogram model. **(C)** Calibration curve of the nomogram model. **(D)** Clinical decision curve of the nomogram model.

To further assess the predictive value of the three clinical prognostic factors identified in the multivariate analyses, we constructed a nomogram model using these variables to predict VNS efficacy in DRE (Figure 2A). ROC analysis showed an area under the curve (AUC) of 0.827 for VNS efficacy (Figure 2B). The calibration curve demonstrated close alignment with ideal performance, indicating a robust predictive capacity for VNS efficacy in DRE (Figure 2C). Additionally, DCA demonstrated a positive net benefit for the predictive model (Figure 2D).

### 3.5 Analysis of the clinical factors of rapid responders to VNS efficacy

**Figure 3:**
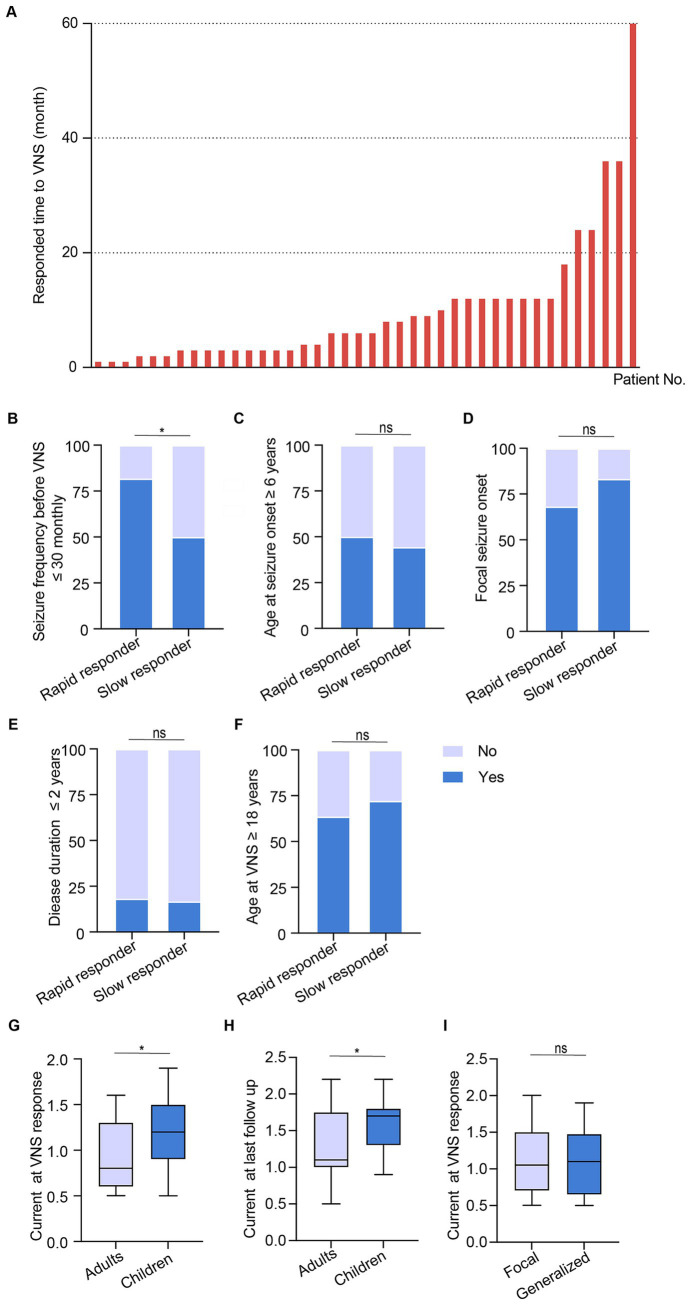
Clinical factors associated with rapid response of VNS in DRE. **(A)** Response time to VNS of the 40 patients with DRE in the responder group. **(B–F)** Comparison between rapid and slow responders, including seizure frequency, age at seizure onset, seizure type, disease duration, and age at VNS. **(G–I)** Differences in VNS current settings among groups: adults vs. children **(G,H)**, focal vs. generalized seizures **(I)**.

As delineated above, 40 (61.5%) patients with DRE, categorized as responders, exhibited a 50–100% reduction in seizure frequency. The response duration ranged from 1 to 60 months, with a median response time of 6 months (Figure 3A). To identify clinical factors associated with rapid response to VNS efficacy, the 40 patients in the responder group were further divided into rapid (response time ≤ 6 months, *n* = 22) and slow responders (response time > 6 months, *n* = 18). We found that only baseline seizure frequency ≤ 30/month was significantly higher in rapid responders than in slow responders (Figure 3B, *P* = 0.033), while no significant differences were observed in other clinical factors between the two groups (Figures 3C–F), indicating the vital role of seizure frequency in VNS efficacy.

Additionally, the VNS current of the VNS responders was collected, and the median VNS current was 1.6 mA (IQR 1.3–1.8, Table 1). The current response to VNS was higher in children than in adults (Figure 3G, *P* = 0.045). Similar results were observed at the last follow-up (Figure 3H, *P* = 0.034). However, no significant differences were found between the focal and generalized seizure groups (Figure 3I, *P* = 0.965).

## 4 Discussion

The current study evaluated 65 patients with epilepsy treated with VNS over a median follow-up of 40 months. The median age at VNS implantation was 9.8 years and the median duration from seizure onset to VNS implantation was 6.0 years, which was lower than the international prospective outcomes registry (CORE-VNS, 10.33 years) (15). Among these, 61.5% patients experienced a 50% reduction in seizure frequency, and 10.8% patients achieved at least 1-year seizure-free status. The average reduction in seizure frequency was 56.7%, consistent with previous studies (16–18). This improvement exceeds that of two randomized controlled trials, possibly due to the longer follow-up in our study (19, 20). A study in Japan, analyzing 362 patients over a 36-month follow-up period, reported median seizure reduction rates of 25.0–66.2% at various time points, with response rates ranging from 38.9 to 58.8% (21). A previous multicenter study with a 5-year follow-up suggested that the responder rate increased from 44.4% in the first year to 64.4% in the fifth year (22). In our study, follow-up extended to 84 months, revealing an increase in VNS efficacy, with seizure reduction rates rising from 35.7% at 6 months to 66.8% at 84 months and response rates increasing from 35.4 to 73.7%. Although participants varied across timepoints in our study, both McHugh classification and responder rates demonstrated a consistent temporal pattern of improving therapeutic efficacy with prolonged VNS duration. These findings suggest that a longer follow-up period may enhance response rates (9), possibly due to the cumulative effects of VNS, extended titration, and concurrent pharmacological adjustments. Notably, the number of ASMs was decreased after the VNS implantation, representing its clinical benefit. Given most patients did not achieve complete seizure freedom, the patients often opt for newer ASMs, ketogenic diets and other interventions in clinical practice, which may increase the seizure reduction and also indicate the VNS efficacy in real-world study. Although randomized controlled trials (RCTs) typically involve shorter follow-up durations and fewer confounding, both long-term retrospective studies and RCTs offer unique benefits and limitations in evaluating VNS efficacy.

Effective titration of VNS current is essential in retrospective studies with long follow-up times. The initial current typically ranges from 0.25–0.5 mA at 30 Hz, with pulse widths of 250 μs or 500 μs. Depending on patient tolerance, the current is gradually increased up to 2.0–2.5 mA or higher (23, 24). In China, VNS devices (PINS) enable incremental adjustments of 0.1 mA, which enhances tolerance, extends the titration period, and prolongs battery life. Due to the immature development of myelin sheaths in the children’s vagus nerve, higher currents may be required for therapeutic response, which was supported in our study. In our paper, the current of the non-responder group was found to be higher than that of the responder group, a detail seldom mentioned in previous research, underscoring the need for increased current for improved efficacy. Previous studies have shown that gradually increasing the current in non-responders leads approximately 20% of them to eventually respond (25). Moreover, 50% of patients do not reach the target current within the first 12 months post-implantation (26), suggesting that the clinical potential of VNS therapy is underutilized and could be enhanced through optimized postoperative management. In addition, variations in VNS programming and device-specific factors in different studies may represent a potential confounding factor in outcome assessments. Similar to pharmacotherapy, optimal stimulation parameters may be inherently constrained by individual patient tolerance thresholds.

In analyzing the clinical factors associated with VNS efficacy in patients with refractory epilepsy, our findings showed that focal seizures predicted a favorable VNS response, aligning with previous studies (27, 28). However, a meta-analysis reported that generalized seizures may yield a better response (29). Another meta-analysis with a larger sample size (5,554 patients) reported no significant difference between focal and generalized seizures (9). Furthermore, Burakgazi et al. (30) found that VNS was more effective in frontal lobe epilepsy than in temporal lobe epilepsy. Nonetheless, in our study, no obvious difference was observed between temporal and non-temporal lobe epilepsy. Additionally, non-lesional cases on MRI demonstrated no association with VNS efficacy compared to lesional cases, while previous studies showed opposite results (9).

Our study encompassed patients with DRE across a wide age range, facilitating age-stratified analysis. Our results indicated that epilepsy onset at or after age six was associated with improved VNS therapeutic outcomes. A meta-analysis indicated that patients with epilepsy onset after the age of 12 years may achieve a higher seizure-freedom rate (11.3% vs. 7.3%) during the 2–4 years postoperative follow-up period (9). A retrospective analysis of 158 DRE patients also demonstrated that age at epilepsy onset ≥ 15 years was significant predictor of VNS response (18). Moreover, for children receiving VNS therapy, a later-onset of epilepsy was associated with better therapeutic outcomes (31), which aligns with our study’s findings. However, another meta-analysis suggested that VNS may be more effective in pediatric patients with epilepsy compared to adults, with response rates of 55.3% in children versus 49.5% in adults (29). Therefore, conclusions regarding the relationship between age at epilepsy onset and VNS efficacy are inconsistent. Nevertheless, our long-term findings favor a later age of epilepsy onset, and a larger sample size and longer follow-up time are urgent to further evaluate related predictors of VNS efficacy in DRE.

The DEEs refer to a group of epilepsies associated with developmental impairment, which may stem from underlying etiology and/or superimposed epileptic activity that causes cognitive and behavioral impairment. Previous studies have suggested VNS therapy for patients with DRE and DEE (31–33). Our univariate analysis showed that the non-responder group had a higher proportion of patients with DEE compared to the responder group (56% vs. 30%), indicating that those with DEE may experience a poorer prognosis with VNS. A study from Norway reported that in patients with DEE, the seizure frequency reduction rates of ≥50% at 6 months and 24 months were 17.1 and 37.1%, respectively, compared to 33.5 and 48.6% in patients without cognitive impairment (34). A study from China found that patients with DEE with milder cognitive impairments exhibited higher response rates to VNS, with all seizure-free patients belonging to this subset (35). Inconsistent results of clinical studies may be associated with the heterogeneity of different patient groups and limited sample size of single-center analysis, highlighting further multicentre studies with larger sample sizes and more clinical data.

Seizure frequency is a vital criterion for evaluating the efficacy of VNS and antiepileptic drug use. In this study, a baseline seizure frequency of < 30/month was confirmed as an effective predictive factor for VNS efficacy. Previous research found that patients experiencing fewer than 20 seizures/month have a better prognosis (36). Riestenberg et al. (18) also demonstrated that baseline seizure frequency < 5/month was significant predictor of VNS response. It’s worth noting that another retrospective study revealed that high seizure frequency and focal interictal epileptiform discharges were potential preoperative predictors of VNS effectiveness (37), but this study focused on the refractory postencephalitic epilepsy (PEE), limiting the VNS curative effect of predictive value for DRE. Our findings revealed that among 40 patients in the responder group, the median response time was 6 months. By this time, patients had already completed the current intensity titration phase (reaching 1.0–1.5 mA over 8–12 weeks) and maintained stable stimulation for at least 3 months. Previous literature also demonstrated that VNS effect at 6 months was a positive predictor of long-term VNS efficacy in DRE, further underscoring the clinical significance of the outcome assessment at 6 months (38). Our results showed that a lower baseline seizure frequency (<30 seizures/month) was associated with a higher likelihood of achieving a response within 6 months. A lower seizure frequency is linked to both higher and faster response rates, a vital finding not previously highlighted in the literature. In summary, patients presenting with a lower baseline seizure frequency exhibited a more rapid and pronounced response to VNS treatment, suggesting that selecting VNS at an earlier stage, when the seizure frequency in DRE patients remains low, may facilitate a quicker therapeutic response and decrease seizure incidence. We hypothesize that the possible mechanism may involve VNS disrupting abnormal neuronal synchronization. Higher seizure frequencies suggest that these patients are more easily “ignited” with more pronounced brain activity synchronization, meaning that VNS requires more time to be effective in these cases, resulting in lower response rates, which was supported by previous literature on EEG (39–41). Previous studies (39–41) have proven that a more significant decrease in the level of synchronization during VNS is strongly correlated with better VNS efficacy, indicating that desynchronization is an important mechanism of VNS.

In addition, this study had some limitations. First, this was a long-term, monocentric, continuous, retrospective study with a limited number of cases spanning a broad age range. Second, while this study aimed to assess seizure improvement and identify predictive factors, providing valuable insights for preoperative screening of future patients with DRE, it did not investigate the improvement of EEG and quality of life outcomes, such as intellectual disability. Third, an easy-to-use nomogram model with ideal performance was constructed to predict VNS efficacy in DRE in the current study. However, no external or multicenter validation was performed to enhance its clinical utility. Further prospective multicenter studies with larger sample sizes and more clinical data are essential to enable personalized assessment and provide guidance on VNS for patients with DRE.

## Data Availability

The raw data supporting the conclusions of this article will be made available by the authors, without undue reservation.
